# Protection of Omicron sub-lineage infection against reinfection with another Omicron sub-lineage

**DOI:** 10.1038/s41467-022-32363-4

**Published:** 2022-08-09

**Authors:** Hiam Chemaitelly, Houssein H. Ayoub, Peter Coyle, Patrick Tang, Hadi M. Yassine, Hebah A. Al-Khatib, Maria K. Smatti, Mohammad R. Hasan, Zaina Al-Kanaani, Einas Al-Kuwari, Andrew Jeremijenko, Anvar Hassan Kaleeckal, Ali Nizar Latif, Riyazuddin Mohammad Shaik, Hanan F. Abdul-Rahim, Gheyath K. Nasrallah, Mohamed Ghaith Al-Kuwari, Adeel A. Butt, Hamad Eid Al-Romaihi, Mohamed H. Al-Thani, Abdullatif Al-Khal, Roberto Bertollini, Laith J. Abu-Raddad

**Affiliations:** 1 Infectious Disease Epidemiology Group, Weill Cornell Medicine-Qatar, Cornell University, Doha, Qatar; 2 World Health Organization Collaborating Centre for Disease Epidemiology Analytics on HIV/AIDS, Sexually Transmitted Infections, and Viral Hepatitis, Weill Cornell Medicine–Qatar, Cornell University, Qatar Foundation – Education City, Doha, Qatar; 3 Department of Population Health Sciences, Weill Cornell Medicine, Cornell University, New York, New York USA; 4 Mathematics Program, Department of Mathematics, Statistics, and Physics, College of Arts and Sciences, Qatar University, Doha, Qatar; 5 Hamad Medical Corporation, Doha, Qatar; 6 Biomedical Research Center, Member of QU Health, Qatar University, Doha, Qatar; 7 Wellcome-Wolfson Institute for Experimental Medicine, Queens University, Belfast, United Kingdom; 8 Department of Pathology, Sidra Medicine, Doha, Qatar; 9 Department of Biomedical Science, College of Health Sciences, Member of QU Health, Qatar University, Doha, Qatar; 10 Department of Public Health, College of Health Sciences, QU Health, Qatar University, Doha, Qatar; 11 Primary Health Care Corporation, Doha, Qatar; 12 Department of Medicine, Weill Cornell Medicine, Cornell University, New York, New York USA; 13 Ministry of Public Health, Doha, Qatar

**Keywords:** Viral infection, Epidemiology, SARS-CoV-2

## Abstract

There is significant genetic distance between SARS-CoV-2 Omicron (B.1.1.529) variant BA.1 and BA.2 sub-lineages. This study investigates immune protection of infection with one sub-lineage against reinfection with the other sub-lineage in Qatar during a large BA.1 and BA.2 Omicron wave, from December 19, 2021 to March 21, 2022. Two national matched, retrospective cohort studies are conducted to estimate effectiveness of BA.1 infection against reinfection with BA.2 (N = 20,994; BA.1-against-BA.2 study), and effectiveness of BA.2 infection against reinfection with BA.1 (N = 110,315; BA.2-against-BA.1 study). Associations are estimated using Cox proportional-hazards regression models after multiple imputation to assign a sub-lineage status for cases with no sub-lineage status (using probabilities based on the test date). Effectiveness of BA.1 infection against reinfection with BA.2 is estimated at 94.2% (95% CI: 89.2–96.9%). Effectiveness of BA.2 infection against reinfection with BA.1 is estimated at 80.9% (95% CI: 73.1–86.4%). Infection with the BA.1 sub-lineage appears to induce strong, but not full immune protection against reinfection with the BA.2 sub-lineage, and vice versa, for at least several weeks after the initial infection.

## Abstract (web summary)

The level of immunity induced by infection with different SARS-CoV-2 Omicron sub-lineages against infection with other sub-lineages is not known. Here, the authors use data from Qatar and show that infection with BA.1 induces strong protection against infection with BA.2, and vice versa, for several weeks.

## Introduction

Reinfections with the severe acute respiratory syndrome coronavirus 2 (SARS-CoV-2) variants that can evade immune response are a concern, potentially challenging the global response to the pandemic^1^. This is especially true of the Omicron^2^ (B.1.1.529) variant and its sub-lineages, of which BA.1 and BA.2 harbor multiple mutations that can mediate immune evasion^2–4^. While SARS-CoV-2 infection with earlier pre-Omicron variants elicits >80% protection against reinfection with the Alpha^2^ (B.1.1.7)^5–8^, Beta^2^ (B.1.351)^5,7,8^, and Delta^2^ (B.1.617.2)^7,9^ variants, immune protection against reinfection with the Omicron BA.1 sub-lineage is inferior at <60%^7^.

**Fig. 1:**
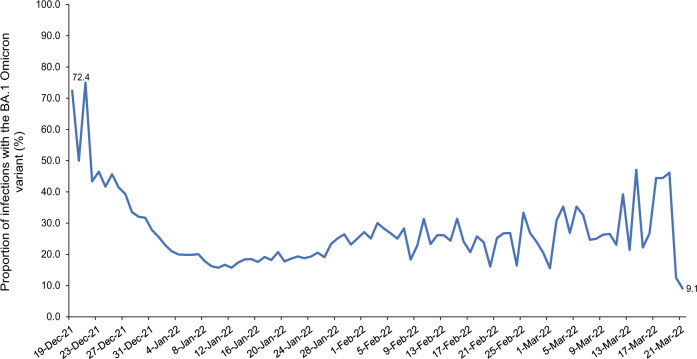
Distribution of SARS-CoV-2 BA.1 versus BA.2 Omicron infections. Proportion of BA.1 (versus BA.2) Omicron infections in PCR-positive tests assessed using TaqPath COVID-19 Combo Kit during the study period.

Qatar experienced a large Omicron wave that started on December 19, 2021 and peaked in mid-January, 2022^7,10–13^. Initially, the BA.1 sub-lineage was predominant, but within days, the BA.2 sub-lineage predominated (Fig. 1). Considering the significant genetic distance between BA.1 and BA.2, we aimed to investigate and estimate immune protection of prior infection with each sub-lineage against the other in Qatar.

We assessed the effectiveness of BA.1 infection against reinfection with BA.2 (BA.1-against-BA.2 study) and the effectiveness of BA.2 infection against reinfection with BA.1 (BA.2-against-BA.1 study) using two matched, retrospective cohort studies (Methods). Individuals in each of the BA.1-infected and BA.2-infected cohorts were exact-matched to uninfected individuals in control cohorts, to control for known differences in the risk of exposure to SARS-CoV-2 infection in Qatar^14–18^.

## Results

### BA.1-against-BA.2 study

**Fig. 2:**
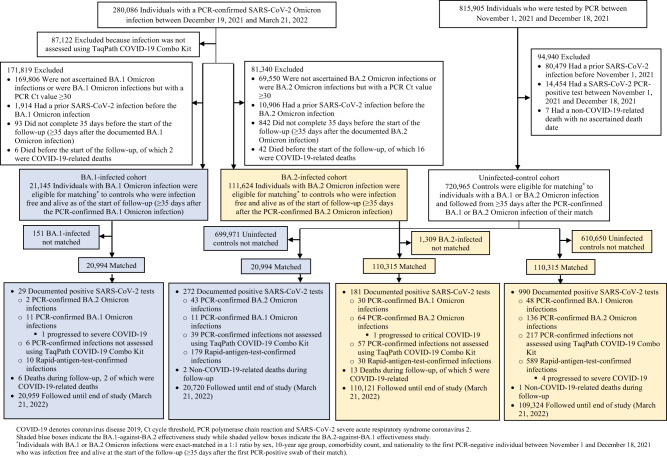
Population selection process. Cohort selection in the BA.1-against-BA.2 and BA.2-against-BA.1 studies.

Figure 2 shows the process for population selection for the BA.1-against-BA.2 study. Table 1 describes the baseline characteristics of the full and matched cohorts. The study was conducted on the total population of Qatar, and thus study population is representative of the internationally diverse but predominantly young and male population of Qatar.

**Table 1:** Baseline characteristics of full and matched cohorts in the BA.1-against-BA.2 and BA.2-against-BA.1 studies

	BA.1-against-BA.2 study						BA.2-against-BA.2 study					
Characteristics	Full eligible cohorts			Matched cohorts^*^			Full eligible cohorts			Matched cohorts^*^		
	BA.1-infected cohort	Uninfected-control cohort	SMD^†^	BA.1-infected cohort	Uninfected-control cohort	SMD^†^	BA.2-infected cohort	Uninfected-control cohort	SMD^†^	BA.2-infected cohort	Uninfected-control cohort	SMD^†^
	*N* = 21,145	*N* = 720,965		*N* = 20,994	*N* = 20,994		*N* = 111,624	*N* = 720,965		*N* = 110,315	*N* = 110,315	
Median age (IQR) — years	33 (25–42)	33 (24–41)	0.08^‡^	33 (25–42)	33 (24–42)	0.00^‡^	34 (26–43)	33 (24–41)	0.16^‡^	34 (26–43)	34 (26–43)	0.00^‡^
**Age group — no. (%)**												
0–19 years	3600 (17.0)	134,856 (18.7)	0.06	3576 (17.0)	3576 (17.0)	0.00	15,805 (14.2)	134,856 (18.7)	0.15	15,588 (14.1)	15,588 (14.1)	0.00
20–29 years	4522 (21.4)	154,465 (21.4)		4488 (21.4)	4488 (21.4)		22,577 (20.2)	154,465 (21.4)		22,301 (20.2)	22,301 (20.2)	
30–39 years	6654 (31.5)	222,959 (30.9)		6624 (31.6)	6624 (31.6)		36,833 (33.0)	222,959 (30.9)		36,513 (33.1)	36,513 (33.1)	
40–49 years	3646 (17.2)	125,106 (17.4)		3622 (17.3)	3622 (17.3)		20,015 (17.9)	125,106 (17.4)		19,844 (18.0)	19,844 (18.0)	
50–59 years	1799 (8.5)	57,487 (8.0)		1782 (8.5)	1782 (8.5)		10,797 (9.7)	57,487 (8.0)		10,648 (9.7)	10,648 (9.7)	
60–69 years	692 (3.3)	20,571 (2.9)		682 (3.3)	682 (3.3)		4051 (3.6)	20,571 (2.9)		3948 (3.6)	3948 (3.6)	
70+ years	232 (1.1)	5,521 (0.8)		220 (1.1)	220 (1.1)		1546 (1.4)	5521 (0.8)		1473 (1.3)	1473 (1.3)	
**Sex**												
Male	10,811 (51.1)	488,631 (67.8)	0.34	10,740 (51.2)	10,740 (51.2)	0.00	65,548 (58.7)	488,631 (67.8)	0.19	64,891 (58.8)	64,891 (58.8)	0.00
Female	10,334 (48.9)	232,334 (32.2)		10,254 (48.8)	10,254 (48.8)		46,076 (41.3)	232,334 (32.2)		45,424 (41.2)	45,424 (41.2)	
**Nationality**^**§**^												
Bangladeshi	328 (1.6)	49,016 (6.8)	0.71	326 (1.6)	326 (1.6)	0.00	3,301 (3.0)	49,016 (6.8)	0.49	3288 (3.0)	3288 (3.0)	0.00
Egyptian	908 (4.3)	29,782 (4.1)		903 (4.3)	903 (4.3)		5465 (4.9)	29,782 (4.1)		5423 (4.9)	5423 (4.9)	
Filipino	2602 (12.3)	39,350 (5.5)		2595 (12.4)	2595 (12.4)		14,048 (12.6)	39,350 (5.5)		13,948 (12.6)	13,948 (12.6)	
Indian	3020 (14.3)	208,042 (28.9)		3012 (14.4)	3012 (14.4)		22,000 (19.7)	208,042 (28.9)		21,895 (19.9)	21,895 (19.9)	
Nepalese	461 (2.2)	52,795 (7.3)		461 (2.2)	461 (2.2)		4732 (4.2)	52,795 (7.3)		4727 (4.3)	4,727 (4.3)	
Pakistani	504 (2.4)	38,329 (5.3)		501 (2.4)	501 (2.4)		3197 (2.9)	38,329 (5.3)		3181 (2.9)	3181 (2.9)	
Qatari	6470 (30.6)	95,364 (13.2)		6404 (30.5)	6,404 (30.5)		26,865 (24.1)	95,364 (13.2)		26,437 (24.0)	26,437 (24.0)	
Sri Lankan	317 (1.5)	19,812 (2.8)		317 (1.5)	317 (1.5)		2945 (2.6)	19,812 (2.8)		2933 (2.7)	2933 (2.7)	
Sudanese	625 (3.0)	12,169 (1.7)		625 (3.0)	625 (3.0)		3330 (3.0)	12,169 (1.7)		3314 (3.0)	3314 (3.0)	
Other nationalities	5910 (28.0)	176,306 (24.5)		5850 (27.9)	5850 (27.9)		25,741 (23.1)	176,306 (24.5)		25,169 (22.8)	25,169 (22.8)	
**Comorbidity count**												
None	15,957 (75.5)	614,763 (85.3)	0.25	15,888 (75.7)	15,888 (75.7)	0.00	84,212 (75.4)	614,763 (85.3)	0.26	83,534 (75.7)	83,534 (75.7)	0.00
1–2	3962 (18.7)	84,178 (11.7)		3908 (18.6)	3908 (18.6)		19,733 (17.7)	84,178 (11.7)		19,362 (17.6)	19,362 (17.6)	
3+	1226 (5.8)	22,024 (3.1)		1198 (5.7)	1198 (5.7)		7679 (6.9)	22,024 (3.1)		7419 (6.7)	7419 (6.7)	

*IQR* interquartile range, *SMD* standardized mean difference.

*Cohorts were matched one-to-one by sex, 10-year age group, nationality, and comorbidity count.

^†^SMD is the difference in the mean of a covariate between groups divided by the pooled standard deviation. An SMD <0.1 indicates adequate matching.

^‡^SMD is for the mean difference between groups divided by the pooled standard deviation.

^§^Nationalities were chosen to represent the most populous groups in Qatar.

^¶^These comprise 131 other nationalities in the BA.1-infected cohort and 202 other nationalities in the uninfected-control cohort in the full eligible cohorts of the BA.1-against-BA.2 study, and 129 other nationalities in the BA.1-infected cohort and 129 other nationalities in the uninfected-control cohort in the matched cohorts of the BA.1-against-BA.2 study. These comprise 156 other nationalities in the BA.2-infected cohort and 202 other nationalities in the uninfected-control cohort in the full eligible cohorts of the BA.2-against-BA.1 study, and 149 other nationalities in the BA.2-infected cohort and 149 other nationalities in the uninfected-control cohort in the matched cohorts of the BA.2-against-BA.1 study.

**Fig. 3:**
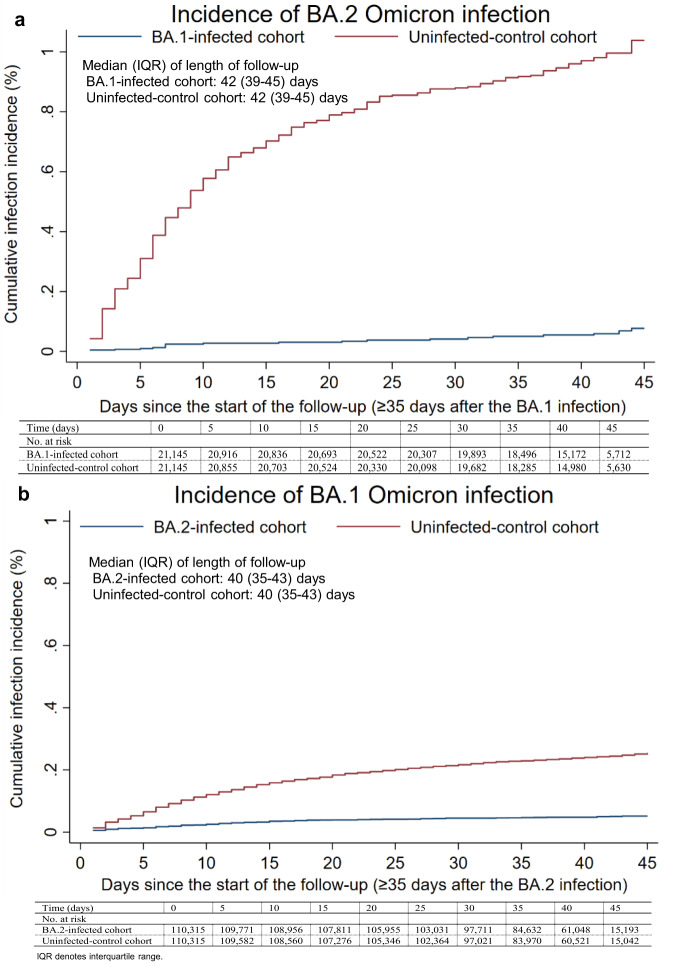
Cumulative incidence of Omicron infections in the BA.1-against-BA.2 and BA.2-against-BA.1 studies. Cumulative incidence of **a** BA.2 and **b** BA.1 Omicron infections in the BA.1-against-BA.2 and BA.2-against-BA.1 studies, respectively. **a** includes 21,145 biologically independent samples for each of the BA.1-infected and the uninfected-control cohorts. **b** includes 110,315 biologically independent samples for each of the BA.2-infected and the uninfected-control cohorts.

The median time of follow-up was 42 days (interquartile range (IQR), 39–45 days) for both the BA.1-infected and the uninfected-control cohorts (Fig. 3). The proportion of individuals who had a polymerase chain reaction (PCR) test or a rapid antigen test (RAT) during follow-up was 24.9% for the BA.1-infected cohort and 27.5% for the uninfected-control cohort. The testing frequency was 0.38 and 0.44 tests per person, respectively (Supplementary Table 1).

Two PCR-documented BA.2 infections, 11 PCR-documented BA.1 infections, 6 other PCR-documented infections, and 10 RAT-documented infections were recorded in the BA.1-infected cohort ≥35 days after the BA.1 infection (Fig. 2). Forty-three PCR-documented BA.2 infections, 11 PCR-documented BA.1 infections, 39 other PCR-documented infections, and 179 RAT-documented infections were recorded during the corresponding time of follow-up for the uninfected-control cohort. Only one PCR-documented BA.1 infection in the BA.1-infected cohort progressed to severe^19^ COVID-19. No other severe^19^, critical^19^, or fatal^20^ COVID-19 cases were recorded. COVID-19 severity is defined in the methods per World Health Organization guidelines^19,20^ (Methods).

In the analysis of the effectiveness of BA.1 infection against reinfection with BA.2 (denoted as PE_S_^BA.1→BA.2^), after using multiple imputations to randomly assign a sub-lineage status for each RAT-documented infection and non-TaqPath PCR-documented infection (Methods), the cumulative incidence of infection was estimated at 0.08% (95% confidence interval (CI): 0.04–0.14%) for the BA.1-infected cohort and at 1.04% (95% CI: 0.89–1.21%) for the uninfected-control cohort, 45 days after the start of follow-up (Fig. 3). The proportion of incident cases assigned through multiple imputations was 85.2% in the BA.1-infected cohort and 79.1% in the uninfected-control cohort.

The hazard ratio for infection, adjusted for sex, 10-year age group, nationality, comorbidity count, and vaccination status, was estimated at 0.06 (95% CI: 0.03–0.11; Table 2). The effectiveness of BA.1 infection against reinfection with BA.2 was estimated at 94.2% (95% CI: 89.2–96.9%).

**Table 2:** Effectiveness against reinfection in the BA.1-against-BA.2 and BA.2-against-BA.1 studies

Epidemiological measure	BA.1-against-BA.2 study		BA.2-against-BA.1 study	
	Effectiveness of BA.1 infection against reinfection with BA.2		Effectiveness of BA.2 infection against reinfection with BA.1	
	Estimate (95% CI)	Effectiveness in % (95% CI)	Estimate (95% CI)	Effectiveness in % (95% CI)
Total follow-up time—BA.1/BA.2-infected cohort (person-weeks)	122,626	—	598,438	—
Total follow-up time—Uninfected-control cohort (person-weeks)	121,572	—	595,257	—
Incidence rate of infection— BA.1/BA.2-infected cohort (per 10,000 person-weeks)	1.1 (0.6 to 2.0)	—	0.9 (0.6 to 1.2)	—
Incidence rate of infection—Uninfected-control cohort (per 10,000 person-weeks)	16.9 (14.6 to 19.7)	—	4.3 (3.7 to 5.1)	—
Unadjusted hazard ratio for infection	0.06 (0.03 to 0.12)	93.6 (88.2 to 96.6)	0.20 (0.15 to 0.29)	79.6 (71.3 to 85.5)
Adjusted hazard ratio for infection^*^	0.06 (0.03 to 0.11)	94.2 (89.2 to 96.9)	0.19 (0.14 to 0.27)	80.9 (73.1 to 86.4)

*CI* confidence interval.

^*^Cox regression analysis adjusted for sex, 10-year age group (Table 1), ten nationality groups (Table 1), comorbidity count (Table 1), and vaccination status. In the BA.1-against-BA.2 study, the adjusted hazard ratio relative to unvaccinated was 1.41 (95% CI: 0.64 to 3.10) for one vaccine dose, 1.92 (95% CI: 1.08 to 3.43) for two vaccine doses, and 1.07 (95% CI: 0.59 to 1.94) for three or more vaccine doses. In the BA.2-against-BA.1 study, the adjusted hazard ratio relative to unvaccinated was 1.74 (95% CI: 0.60 to 5.00) for one vaccine dose, 1.84 (95% CI: 1.19 to 2.87) for two vaccine doses, and 1.31 (95% CI: 0.85 to 2.01) for three or more vaccine doses.

In a sensitivity analysis adjusting the regression for time since vaccination in addition to vaccination status, the adjusted hazard ratio for infection was estimated at 0.06 (95% CI: 0.03–0.11), and the effectiveness was estimated at 94.1% (95% CI: 89.1–96.8%).

In a sensitivity analysis adjusting the hazard ratio by the ratio of testing frequencies, the adjusted hazard ratio for infection was estimated at 0.07 (95% CI: 0.03–0.13), and the effectiveness was estimated at 93.1% (95% CI: 87.3–96.5%).

### BA.2-against-BA.1 study

Figure 2 shows the process for population selection for the BA.2-against-BA.1 study. Table 1 describes the baseline characteristics of the full and matched cohorts. The study was conducted on the total population of Qatar, and thus study population is representative of the population of Qatar.

The median time of follow-up was 40 days (IQR, 35–43 days) for both the BA.2-infected cohort and the uninfected-control cohort (Fig. 3). The proportion of individuals who had a PCR or RAT test during follow-up was 20.2% for the BA.2-infected cohort and 22.8% for the uninfected-control cohort. The testing frequency was 0.30 and 0.35 tests per person, respectively (Supplementary Table 1).

Thirty PCR-documented BA.1 infections, 64 PCR-documented BA.2 infections, 57 other PCR-documented infections, and 30 RAT-documented infections were recorded in the BA.2-infected cohort ≥35 days after the BA.2 infection (Fig. 2). Forty-eight PCR-documented BA.1 infections, 136 PCR-documented BA.2 infections, 217 other PCR-documented infections, and 589 RAT-documented infections were recorded during the corresponding time of follow-up for the uninfected-control cohort. Only one PCR-documented BA.2 infection in the BA.2-infected cohort progressed to critical^19^ COVID-19. Meanwhile, 4 RAT-documented infections in the uninfected-control cohort progressed to severe^19^ COVID-19. No other severe^19^, critical^19^, or fatal^20^ COVID-19 cases were recorded.

In the analysis of the effectiveness of BA.2 infection against reinfection with BA.1 (denoted as PE_S_^BA.2→BA.1^), after using multiple imputations to randomly assign a sub-lineage status for each RAT-documented infection and non-TaqPath PCR-documented infection (Methods), the cumulative incidence of infection was estimated at 0.05% (95% CI: 0.04–0.07%) for the BA.2-infected cohort and at 0.25% (95% CI: 0.21–0.30%) for the uninfected-control cohort, 45 days after the start of follow-up (Fig. 3). The adjusted hazard ratio for infection was estimated at 0.19 (95% CI: 0.14–0.27; Table 2). The effectiveness of BA.2 infection against reinfection with BA.1 was estimated at 80.9% (95% CI: 73.1–86.4%). The proportion of incident cases assigned through multiple imputations was 44.3% in the BA.2-infected cohort and 81.2% in the uninfected-control cohort.

In a sensitivity analysis adjusting the regression for time since vaccination in addition to vaccination status, the adjusted hazard ratio for infection was estimated at 0.19 (95% CI: 0.14–0.27), and the effectiveness was estimated at 80.9% (95% CI: 73.1–86.4%).

In a sensitivity analysis adjusting the hazard ratio by the ratio of testing frequencies, the adjusted hazard ratio for infection was estimated at 0.22 (95% CI: 0.16–0.32), and the effectiveness was estimated at 77.8% (95% CI: 62.7–81.3%).

## Discussion

Reinfections with BA.2 (or BA.1) shortly after infection with BA.1 (or BA.2) have been observed in Qatar during a large Omicron wave in which both sub-lineages were intensely circulating. However, it is remarkable that the incidence of reinfection, regardless of sub-lineage, was much lower in the BA.1-infected and BA.2-infected cohorts than the incidence of infection in the corresponding uninfected-control cohorts (Fig. 2), consistent with strong immune protection against reinfection regardless of sub-lineage. Our findings indicate that infection with an Omicron sub-lineage appears to elicit strong protection against reinfection with the other sub-lineage at effectiveness that exceeds 80%, similar to the protection observed for infection against reinfection with an original virus or earlier pre-Omicron variants (Alpha, Beta, or Delta)^5–9,21,22^.

These findings, in the context of broader evidence for natural immunity^5–9,21,23,24^, suggest that natural immunity of SARS-CoV-2 variants cluster into two groups: pre-Omicron variants and Omicron sub-lineages. Within each group, there appears to be strong protection against reinfection with effectiveness that exceeds 80%. However, across groups, the protection may not exceed 60%, as was observed recently^7^. This conclusion is also supported by evidence of the sensitivity of variants to SARS-CoV-2 antibodies^2–4,25,26^.

This study has limitations. Since the Omicron wave was initially dominated by BA.1 (Fig. 1), the follow-up in the BA.2-against-BA.1 study was shifted in calendar time to after the follow-up in the BA.1-against-BA.2 study. With the high intensity of infection transmission, followed by the rapid decline of the Omicron wave, more of the uninfected controls in the BA.2-against-BA.1 study may have experienced an undocumented Omicron infection compared to the uninfected controls in the BA.1-against-BA.2 study. This would bias PE_S_^BA.2→BA.1^ to a lower value and may explain why PE_S_^BA.2→BA.1^ was lower than that of PE_S_^BA.1→BA.2^.

Effectiveness against reinfection was estimated for only a few weeks after the primary infection. A longer duration of follow-up may identify differences not yet seen given the recency of the Omicron wave. However, evidence has been consistent that natural immunity, unlike vaccine immunity, wanes slowly with minimal waning for at least several months after primary infection^5–9,12,21,23,24^.

BA.1 and BA.2 ascertainment was based on proxy criteria, presence or absence of an S-gene “target failure” (SGTF) case using the TaqPath PCR assay, but this method of ascertainment is well established not only for Omicron sub-lineages, but also for other variants such as Alpha^6,27,28^. BA.1 and BA.2 ascertainment was not possible for infections diagnosed using RAT or other PCR testing. This limitation was mitigated through multiple imputations using the information on the distribution of known BA.1 and BA.2 cases for each calendar day and pooling estimates following Rubin’s rules^29,30^ (Methods). This approach allowed us to factor statistically the uncertainty in classifying each positive test where BA.1/BA.2 ascertainment was not possible. Even after accounting for this uncertainty in our estimates, the corresponding 95% confidence intervals were relatively narrow, affirming the findings of this study.

An alternative analysis plan is to disregard the RAT results except for censoring. However, RAT testing was not uniform throughout the Omicron wave and started just before the peak of the Omicron wave. Also, the BA.1 and BA.2 sub-waves overlapped but were still shifted in calendar time (Fig. 1). Furthermore, although the BA.1-infected and BA.2-infected cohorts were strongly protected against reinfection during follow-up, at the time when RAT testing was being scaled up, the control cohorts were not, leading to the higher rates of RAT-positive tests among the control cohorts. These factors can introduce serious bias if we are to disregard the RAT results, as noted also in the differences in the distribution of RAT-diagnosed infections in the BA.1-infected cohort versus the BA.2-infected cohort (Fig. 2).

With the large Omicron wave in Qatar, the use of RAT testing was implemented broadly as a replacement for PCR testing, as PCR testing capacity was not sufficient to handle the demand. The distribution of BA.1 versus BA.2 in any specific calendar day should thus not differ by testing modality (PCR or RAT). Testing, regardless of modality, was implemented because of the appearance of symptoms or for other routine reasons; it was not dependent on the sub-lineage to bias the results. Our multiple imputation approach randomly assigned a sub-lineage status (BA.1 or BA.2) for each RAT-documented infection and non-TaqPath PCR-documented infection diagnosed on a specific calendar day, using the information on the probability of the infection being BA.1 or BA.2 in that specific day. This probability was determined by the observed distribution of identified BA.1 and BA.2 infections on each calendar day (Fig. 1).

Most of the incident cases were assigned through this multiple imputation approach. The approach implicitly assumes that, given the observed data, the reason for the missing data does not depend on the unseen data^31^. While no alternative analyses were conducted, such as a weighting approach^31^, we believe this is a valid assumption, as testing, regardless of modality, was implemented because of the appearance of symptoms or for other routine reasons, and it should not depend on the specific sub-lineage. It is worth stressing here that the findings of this study of strong immune protection against reinfection are evident even if we could not ascertain the BA.1/BA.2 status of any infection, regardless of the implemented approach of multiple imputations. Only 29 infections were recorded in the BA.1-infected cohort versus 272 infections in the uninfected-control cohort (Fig. 2). Similarly, only 181 infections were recorded in the BA.2-infected cohort versus 990 infections in the uninfected-control cohort (Fig. 2). The protection against reinfection was strong regardless of BA.1/BA.2 ascertainment.

Some Omicron infections may have been misclassified as Delta infections, but Delta incidence was limited during the time of follow-up (Methods). The presence of Delta infections may have led to the underestimation of immune protection against BA.1 and BA.2, as protection of BA.1 or BA.2 against Delta is perhaps lower than that against Omicron sub-lineages^7,12^. With the recency of the Omicron wave, we had to use a short interval of 35 days to define reinfection, possibly introducing bias due to the misclassification of prolonged infections as reinfections. However, such bias is less likely to affect PE_S_^BA.1→BA.2^ and PE_S_^BA.2→BA.1^, but may affect estimates of the effectiveness of BA.1 (or BA.2) infection against reinfection with BA.1 (or BA.2); that is, when both the primary infection and the reinfection are both due to the same sub-lineage. Such effectiveness estimates are not reported in this study (but found in a separate analysis to be comparable to PE_S_^BA.1→BA.2^ and PE_S_^BA.2→BA.1^). Regardless, such bias could underestimate immune protection, as it would inflate incident cases only in the BA.1-infected or BA.2-infected cohorts, but not in the control cohorts, thereby further supporting our findings of strong protection against reinfection.

Persons with a record of a prior infection were excluded, but not persons with a record of prior vaccination. Vaccine coverage is high in Qatar at about 90% for two-dose (primary series) vaccination^32^. Excluding vaccinated persons would have rendered the cohorts too small for a meaningful analysis. However, we adjusted our estimates for vaccination status in the regression model. Since vaccine effectiveness^12^ and durability^13^ against each of BA.1 and BA.2 are comparable, our approach for controlling for vaccine immunity is not likely to have been biased by differential effects of vaccination against each of BA.1 and BA.2.

Evidence indicates a rapid waning in vaccine protection over time^12,13,33–35^. However, sensitivity analyses adjusting for time since vaccination in addition to vaccination status confirmed the same findings. Although differences in SARS-CoV-2 testing frequency across the infected and uninfected cohorts were noted, these differences were relatively small. Moreover, adjustment for the differences in testing frequency in sensitivity analyses confirmed similar findings. The travel history of cohort members was not available. However, there is no reason to believe that travel or leaving the country could have differentially affected the followed matched cohorts to affect our results. Of note that both case and control cohorts were defined on the basis of recent PCR tests to ensure that all persons in these cohorts have a record of a recent active residence in Qatar (Methods).

Temporal effects are unlikely to affect our study. The matched cohorts were followed over the same calendar time. The study findings are unlikely to be explained by each sub-lineage being transmitted in different sub-communities or population strata within Qatar. Qatar is a small country and is essentially a city-state where 89% of the population are expatriates from over 150 countries coming for employment^14^. About 60% of the population are men and young craft and manual workers working in development projects^14,36^. Nationality, age, and sex provide a powerful proxy for socio-economic status in this country^14–18,36^. Nationality alone is strongly associated with occupation^14–18,36^. Infection incidence was broadly distributed across neighborhoods/areas. Matching was also done to control for demographic and socio-economic factors known to affect infection exposure in Qatar^14–18^. Having said so, BA.2, compared to BA.1, affected more the predominantly men craft and manual worker population where seroprevalence levels are highest^14–18,36^, as suggested by the different sex ratios for BA.1 versus BA.2 infections (Table 1). This appears to be due to BA.2’s higher infectiousness that allowed it to reach more susceptibles within this population segment^37^.

RAT testing has lower sensitivity and specificity than PCR testing^38^. However, the RATs in use in Qatar have high sensitivity and specificity, are of global use and have been validated (Methods). The RAT testing rates were similar for both the case and control cohorts (Supplementary Table 1). False positivity or false negativity can affect both case and control cohorts, not one of them. Therefore, it is not likely that the use of RAT testing may have biased our estimates. Yet, it is unknown whether there are differences in the sensitivity and specificity of RATs by sub-lineage statuses, such as due to the higher viral load of BA.2 infections compared to BA.1 infections^37^.

As an observational study, the investigated cohorts were neither blinded nor randomized, so unmeasured or uncontrolled confounding cannot be excluded. While matching was done for sex, age, nationality, and comorbidity count, this was not possible for other factors, such as occupation, as such data were not available. However, matching was done to control for factors that affect infection exposure in Qatar^14–18^. Matching by nationality may have partially controlled for differences in occupational risk or socio-economic status, given the association between nationality and occupation in Qatar^14–18^. Lastly, matching by the considered factors has been shown to provide adequate control of bias in studies that used control groups in Qatar to test for null effects^33,39–42^. These control groups included unvaccinated cohorts versus vaccinated cohorts within two weeks of the first dose^33,39–41^, when vaccine protection is negligible^43,44^, and mRNA-1273- versus BNT162b2-vaccinated cohorts, also in the first two weeks after the first dose^42^. A strength of this study is the exclusion of those with a documented prior infection before the Omicron wave to minimize potential confounding introduced by natural immunity due to earlier pre-Omicron variants.

In conclusion, infection with an Omicron sub-lineage appears to induce strong but not full immune protection against reinfection with the other sub-lineage, for at least several weeks after the initial infection.

## Methods

### Data sources

This study analyzed the national, federated databases for coronavirus disease 2019 (COVID-19), retrieved from the integrated nationwide digital-health information platform. Databases include all severe acute respiratory syndrome coronavirus 2 (SARS-CoV-2)-related data and associated demographic information, with no missing information, since pandemic onset. These include all polymerase chain reaction (PCR) testing and, more recently, rapid antigen testing (RAT) conducted at healthcare facilities (from January 5, 2022 onwards). Testing in Qatar is done at a mass scale and mostly for routine reasons^12,33^. About 75% of those diagnosed are diagnosed not because of the appearance of symptoms but because of routine testing^12,33^. All testing done during follow-up in the present study was included in the analysis.

The databases also include all COVID-19 vaccination records, COVID-19 hospitalizations, infection severity and mortality classifications per World Health Organization (WHO) guidelines^19,20^, in addition to sex, age, nationality, and comorbidity information retrieved from the national registry. Further description of these national databases can be found in previous publications^5,11,14,33,45,46^.

### Study design

We assessed the effectiveness of BA.1 infection against reinfection with BA.2 (denoted as PE_S_^BA.1→BA.2^; BA.1-against-BA.2 study) and the effectiveness of BA.2 infection against reinfection with BA.1 (denoted as PE_S_^BA.2→BA.1^; BA.2-against-BA.1 study), using two matched, retrospective cohort studies. *PE*_*S*_ was defined as the proportional reduction in susceptibility to documented infection, regardless of symptoms, among those with the prior sub-lineage infection versus those without^7,8^. Informed by viral genome sequencing and real-time reverse-transcription PCR (RT-qPCR) genotyping, a SARS-CoV-2 infection with the BA.1 sub-lineage was proxied as an S-gene “target failure” (SGTF) case using the TaqPath COVID-19 Combo Kit (Thermo Fisher Scientific, USA)^27^. Conversely, an infection with the BA.2 sub-lineage was proxied as a non-SGTF case using the same assay.

The BA.1-against-BA.2 study followed a cohort of individuals with documented BA.1 infections and compared the incidence of BA.2 infection in this cohort with that in a control cohort of individuals with no record of prior SARS-CoV-2 infection. The BA.2-against-BA.1 study followed a cohort of individuals with documented BA.2 infections and compared the incidence of BA.1 infection in this cohort with that in a control cohort of individuals with no record of prior SARS-CoV-2 infection.

To optimize specificity in defining the cohorts, the BA.1-infected and BA.2-infected cohorts were defined based on the existence of an infection documented only using PCR and with a PCR cycle threshold value <30, between December 19, 2021 and March 21, 2022 (the date when PCR testing using the TaqPath COVID-19 Combo Kit that targets the S-gene was discontinued). In all cohorts of the two studies, the two case and two control cohorts, persons with a record of a prior infection before December 19, 2021 were excluded. This is to ensure that estimated PE_S_^BA.1→BA.2^ and PE_S_^BA.2→BA.1^ are not affected by immunity induced by prior infections with earlier variants. Record of COVID-19 vaccination was not an exclusion criterion, but the regression analyses adjusted for vaccination status (unvaccinated, one dose, two doses, or three or more doses at the start of the follow-up). The control cohorts in the two studies were defined on the basis of PCR-negative tests between November 1, 2021 and December 18, 2021 (Fig. 2), to ensure that all persons in these cohorts have a record of a recent active residence in Qatar.

Ideally, SARS-CoV-2 reinfection is defined as a documented infection ≥90 days after an earlier infection to avoid misclassification of prolonged infections as reinfections if a shorter time interval is used^21,23,24^. Since the Omicron wave started only recently, this definition could not be used. Analysis of cohort sizes and durations of follow-up was conducted to identify the longest time interval possible while maintaining adequate cohort size, durations of follow-up, and precision of estimates. Informed by this analysis of the implications of using different time intervals, reinfection was defined as a documentation of infection ≥35 days after the prior infection. For example, if we are to set the time interval at 90 days instead of 35 days, the size of the cohort would have been reduced (before matching) from 21,145 individuals to only 36 individuals in the BA.1-against-BA.2 study and from 111,624 individuals to only 20 individuals in the BA.2-against-BA.1 study. Moreover, at the 35-day interval, only a small number of documented reinfections could have been prolonged prior infections rather than true reinfections^21–24,47^. Cohorts were thus followed after completion of 35 days since documentation of the BA.1 (or BA.2) infection.

Individuals in each of the BA.1-infected and BA.2-infected cohorts were exact-matched in a 1:1 ratio by sex, 10-year age group, nationality, and comorbidity count (none, one, two, three, or more comorbidities) to uninfected individuals in control cohorts (Fig. 2), to control for known differences in the risk of exposure to SARS-CoV-2 infection in Qatar^14–18^. Matching was performed through an iterative process that ensured that each control was alive and infection-free at the start of follow-up. Follow-up was defined, for each matched pair, at ≥35 days after documentation of the BA.1 infection in the BA.1-infected cohort and documentation of the BA.2 infection in the BA.2-infected cohort. Cohorts were followed up until the first of the following events: a PCR-documented BA.1 infection, a PCR-documented BA.2 infection, and other PCR-documented infection (documented with an assay other than TaqPath), RAT-documented infection, death, and end of study censoring (March 21, 2022).

### COVID-19 severity, criticality, and fatality classification

Classification of COVID-19 case severity (acute-care hospitalizations)^19^, criticality (intensive-care-unit hospitalizations)^19^, and fatality^20^ followed WHO guidelines, and assessments were made by trained medical personnel using individual chart reviews. Each person who had a PCR-positive test result and COVID-19 hospital admission was subject to an infection severity assessment every three days until discharge or death, regardless of the hospital stay length or the time between the PCR-positive test and the final disease outcome. Individuals who progressed to severe^19^, critical^19^, or fatal^20^ COVID-19 between the PCR-positive test result and the end of the study were classified based on their worst outcome, starting with death, followed by critical disease, and then severe disease.

WHO defines severe COVID-19 as a SARS-CoV-2 infected individual with “oxygen saturation of <90% on room air, and/or respiratory rate of >30 breaths/minute in adults and children >5 years old (or ≥60 breaths/minute in children <2 months old or ≥50 breaths/minute in children 2–11 months old or ≥40 breaths/minute in children 1–5 years old), and/or signs of severe respiratory distress (accessory muscle use and inability to complete full sentences, and, in children, very severe chest wall indrawing, grunting, central cyanosis, or presence of any other general danger signs)”^19^. Detailed criteria are in the WHO technical report^19^.

Critical COVID-19 is defined as a SARS-CoV-2 infected individual with “acute respiratory distress syndrome, sepsis, septic shock, or other conditions that would normally require the provision of life-sustaining therapies such as mechanical ventilation (invasive or non-invasive) or vasopressor therapy”^19^. Detailed criteria are in the WHO technical report^19^.

COVID-19 death is defined as “a death resulting from a clinically compatible illness, in a probable or confirmed COVID-19 case, unless there is a clear alternative cause of death that cannot be related to COVID-19 disease (e.g., trauma). There should be no period of complete recovery from COVID-19 between illness and death. Death due to COVID-19 may not be attributed to another disease (e.g., cancer) and should be counted independently of preexisting conditions that are suspected of triggering a severe course of COVID-19”. Detailed criteria are in the WHO technical report^20^.

### Laboratory methods

#### Real-time reverse-transcription polymerase chain reaction testing

Nasopharyngeal and/or oropharyngeal swabs were collected for PCR testing and placed in Universal Transport Medium (UTM). Aliquots of UTM were: (1) extracted on KingFisher Flex (Thermo Fisher Scientific, USA), MGISP-960 (MGI, China), or ExiPrep 96 Lite (Bioneer, South Korea), followed by testing with RT-qPCR using TaqPath COVID-19 Combo Kits (Thermo Fisher Scientific, USA) on an ABI 7500 FAST (Thermo Fisher Scientific, USA); (2) tested directly on the Cepheid GeneXpert system using the Xpert Xpress SARS-CoV-2 (Cepheid, USA); or (3) loaded directly into a Roche cobas 6800 system and assayed with the cobas SARS-CoV-2 Test (Roche, Switzerland). The first assay targets the viral S, N, and ORF1ab gene regions. The second targets the viral N and E-gene regions, and the third targets the ORF1ab and E-gene regions.

All PCR testing was conducted at the Hamad Medical Corporation Central Laboratory or Sidra Medicine Laboratory, following standardized protocols.

#### Rapid antigen testing

SARS-CoV-2 antigen tests were performed on nasopharyngeal swabs using one of the following lateral flow antigen tests: Panbio COVID-19 Ag Rapid Test Device (Abbott, USA; sensitivity: 91.4%, specificity: 99.8%);^48^ SARS-CoV-2 Rapid Antigen Test (Roche, Switzerland; sensitivity: 95.5%, specificity: 99.2%);^49^ Standard Q COVID-19 Antigen Test (SD Biosensor, Korea; sensitivity: 90.7%, specificity: 98.9%);^50^ or CareStart COVID-19 Antigen Test (Access Bio, USA; sensitivity: 93.8%, specificity: 99.3%)^51^. All antigen tests were performed point-of-care according to each manufacturer’s instructions at public or private hospitals and clinics throughout Qatar with prior authorization and training by the Ministry of Public Health (MOPH). Antigen test results were electronically reported to the MOPH in real-time using the Antigen Test Management System, which is integrated with the national COVID-19 database.

#### Classification of infections by variant type

Surveillance for SARS-CoV-2 variants in Qatar is mainly based on viral genome sequencing and multiplex RT-qPCR variant screening^52^ of random positive clinical samples^7,10–13,33,40,53–55^, complemented by deep sequencing of wastewater samples^10,56^. During this study, from December 19, 2021 to March 21, 2022, infection incidence was vastly dominated by the Omicron variant.

A total of 315 random SARS-CoV-2-positive specimens collected between December 19, 2021 and January 22, 2022 were viral whole-genome sequenced on a Nanopore GridION sequencing device. Of these, 300 (95.2%) were confirmed as Omicron infections and 15 (4.8%) as Delta (B.1.617.2)^2^ infections^10–13^. Of 286 Omicron infections with confirmed sub-lineage status, 68 (23.8%) were BA.1 cases and 218 (76.2%) were BA.2 cases.

Additionally, a total of 8811 random SARS-CoV-2-positive specimens collected between December 22, 2021 and February 28, 2022 were RT-qPCR genotyped. The RT-qPCR genotyping identified 470 B.1.617.2-like Delta case, 1017 BA.1-like Omicron cases, 4429 BA.2-like Omicron cases, and 2895 were undetermined cases where the genotype could not be assigned due to weak PCR Ct values.

The accuracy of the RT-qPCR genotyping was verified against either Sanger sequencing of the receptor-binding domain (RBD) of the SARS-CoV-2 surface glycoprotein (S) gene or by viral whole-genome sequencing on a Nanopore GridION sequencing device. From 147 random SARS-CoV-2-positive specimens, all collected in December of 2021, RT-qPCR genotyping was able to assign a genotype in 129 samples. The agreement between RT-qPCR genotyping and sequencing was 100% for Delta (*n* = 82), 100% for Omicron BA.1 (*n* = 18), and 100% for Omicron BA.2 (*n* = 29). Of the remaining 18 specimens, ten failed PCR amplification and sequencing and eight could not be assigned a genotype by RT-qPCR (four of eight were B.1.617.2 by sequencing, and the remaining four failed sequencing). All the variant RT-qPCR genotyping was conducted at the Sidra Medicine Laboratory following standardized protocols.

The large Omicron-wave exponential-growth phase in Qatar started on December 19, 2021 and peaked in mid-January, 2022^7,10–13^. The study duration coincided with the intense Omicron wave where Delta incidence was limited. Accordingly, any PCR or RAT-positive test during the study duration, between December 19, 2021 and March 21, 2022, was assumed to be an Omicron infection.

### Statistical analysis

Full and matched cohorts were described using frequency distributions and measures of central tendency. Group comparisons were performed using standardized mean differences (SMDs), with an SMD <0.1 indicating adequate matching^57^.

Due to the large Omicron wave, the use of RAT testing was expanded rapidly to supplement PCR testing starting from January 5, 2022, precluding ascertainment of the Omicron sub-lineage in these tests. While 69.6% of all PCR tests (positive or negative) during the study were conducted using an assay that targets the S-gene, a minority of infections were documented with other commercial PCR kits/platforms that are not affected by the del69/70 mutation in the S-gene (Laboratory methods), also precluding ascertainment of the Omicron sub-lineage in these tests (Fig. 2).

To avoid potential bias introduced by missing data, multiple imputations with 100 iterations was implemented to randomly assign a sub-lineage status (BA.1 or BA.2) for each RAT-documented infection and non-TaqPath PCR-documented infection diagnosed on a specific calendar day, using the information on the probability of the infection being BA.1 or BA.2 in that specific day. This probability was determined by the observed distribution of identified BA.1 and BA.2 infections on each calendar day (Fig. 1) after applying a 3-day moving average to smoothen the distribution curve.

For each of the 100 generated datasets, the cumulative incidence of infection was estimated in each cohort using the Kaplan–Meier estimator method^58^. Cumulative incidence of infection was defined as the proportion of individuals at risk whose primary endpoint was an incident infection during follow-up. The incidence rate of infection in each cohort, which was defined as the number of identified infections divided by the number of person-weeks contributed by all individuals in the cohort, was estimated using a Poisson log-likelihood regression model with the STATA 17.0 stptime command. The hazard ratio comparing the incidence of infection in case versus control cohorts was calculated using Cox regression adjusted for matching factors and COVID-19 vaccination status (unvaccinated, one dose, two doses, or three or more doses at the start of the follow-up) with the STATA 17.0 stcox command. Shoenfeld residuals and log-log plots for survival curves were used to test the proportional-hazards assumption and to investigate its adequacy.

Estimates for the cumulative incidence of infection, the incidence rate of infection, hazard ratio, and corresponding standard errors across the 100 datasets were then log-transformed and pooled following Rubin’s rules^29,30^, prior to back-transformation. Pooled standard errors were used to derive 95% confidence intervals (CIs). PE_S_^BA.1→BA.2^ and PE_S_^BA.2→BA.1^ were estimated using the equation: Effectiveness = 1-pooled adjusted hazard ratio.

Sensitivity analyses were conducted by adjusting the Cox regression for time since vaccination at the start of the follow-up, in addition to vaccination status (that is, the categories are unvaccinated, one dose, two doses ≤6 months, two doses >6 months, three doses ≤2 months, or three doses >2 months). Sensitivity analyses were further conducted by adjusting the estimates for differences in testing frequency between the cohorts. Statistical analyses were conducted in Stata/SE version 17.0^59^.

### Oversight

Hamad Medical Corporation and Weill Cornell Medicine-Qatar Institutional Review Boards approved this retrospective study with a waiver of informed consent (approval references: MRC-05-011 and 20-00017, respectively). The study was reported following Strengthening the Reporting of Observational Studies in Epidemiology (STROBE) guidelines. The STROBE checklist is found in Supplementary Table 2.

### Reporting summary

Further information on research design is available in the Nature Research Reporting Summary linked to this article.

## Supplementary information

Supplementary Information

Reporting Summary

## Data Availability

The dataset of this study is a property of the Qatar Ministry of Public Health that was provided to the researchers through a restricted-access agreement that prevents sharing the dataset with a third party or publicly. The data are available under restricted access for the preservation of confidentiality of patient data. Access can be obtained through a direct application for data access to Her Excellency the Minister of Public Health (https://www.moph.gov.qa/english/OurServices/eservices/Pages/Governmental-Health-Communication-Center.aspx). The raw data are protected, and are not available due to data privacy laws. Data were available to authors through.csv files where information has been downloaded from the CERNER database system (no links/accession codes were available to authors). Aggregate data are available within the manuscript and its Supplementary information.

## Code availability

Standard epidemiological analyses were conducted using standard commands in STATA/SE 17.0^59^. The code for pooling multiple imputed datasets can be found at: https://www.pharmasug.org/proceedings/2017/SP/PharmaSUG-2017-SP05.pdf^30^.
