# Supplementary material for: Protection of Omicron sub-lineage infection against reinfection with another Omicron sub-lineage
Source: Nat Commun. 2022 Aug 9;13:4675. doi: 10.1038/s41467-022-32363-4 (PMC9362989; doi:10.1038/s41467-022-32363-4)
Supplement: Supplementary file 1 — Supplementary Information [file 41467_2022_32363_MOESM1_ESM.pdf]

Supplementary Appendix

Supplementary Table 1. Distribution of tests by reason for testing. Distribution of all tests, positive or negative, during follow-up by reason for testing.

Reason for testing	BA.1-infected cohort	Uninfected-control cohort	SMD*	BA.2-infected cohort	Uninfected-control cohort	SMD*
PCR testing	N tests=2,780	N tests=3,206		N tests=11,322	N tests=13,131	
Clinical suspicion	120 (4.3)	104 (3.2)	0.29	535 (4.7)	510 (3.9)	0.24
Contact tracing	172 (6.2)	469 (14.6)		627 (5.5)	1,420 (10.8)	
Survey	305 (11.0)	374 (11.7)		1,875 (16.6)	1,526 (11.6)	
Individual request	49 (1.8)	50 (1.6)		267 (2.4)	259 (2.0)	
Travel-related testing	2,062 (74.2)	2,126 (66.3)		7,591 (67.1)	8,980 (68.4)	
Healthcare routine testing	59 (2.1)	69 (2.2)		347 (3.1)	376 (2.9)	
Other	13 (0.5)	14 (0.4)		80 (0.7)	60 (0.5)	
Rapid antigen testing	N tests=5,281	N tests=6,134		N tests=21,261	N tests=25,682	
Clinical suspicion	89 (1.7)	94 (1.5)	0.16	287 (1.4)	399 (1.6)	0.08
Contact tracing	12 (0.2)	19 (0.3)		109 (0.5)	121 (0.5)	
Survey	64 (1.2)	83 (1.4)		244 (1.2)	304 (1.2)	
Individual request	606 (11.5)	694 (11.3)		2,272 (10.7)	2,800 (10.9)	
Travel-related testing	1,558 (29.5)	1,421 (23.2)		5,622 (26.4)	5,946 (23.2)	
Healthcare routine testing	257 (4.9)	417 (6.8)		1,310 (6.2)	1,745 (6.8)	
Other	1 (0.02)	0 (0.0)		2 (0.01)	2 (0.01)	
No reason specified†	2,694 (51.0)	3,406 (55.5)		11,415 (53.7)	14,365 (55.9)	

Abbreviations: PCR, polymerase chain reaction; SMD, standardized mean difference.

*SMD is the difference in the mean of a covariate between groups divided by the pooled standard deviation. An SMD <0.1 indicates adequate matching/balance.

†With the large Omicron wave, use of rapid antigen testing was expanded suddenly and rapidly to supplement PCR testing, but reason for testing was not included in these tests till few weeks later.

Supplementary Table 2. Strengthening the Reporting of Observational Studies in Epidemiology (STROBE) checklist. STROBE checklist for cohort studies.

	Item No	Recommendation	Main Text page
Title and abstract	1	(*a*) Indicate the study's design with a commonly used term in the title or the abstract	Abstract
		(*b*) Provide in the abstract an informative and balanced summary of what was done and what was found	Abstract
Introduction			
Background/rationale	2	Explain the scientific background and rationale for the investigation being reported	Introduction
Objectives	3	State specific objectives, including any prespecified hypotheses	Introduction
Methods			
Study design	4	Present key elements of study design early in the paper	Introduction, paragraph 3 & Methods ('Study design')
Setting	5	Describe the setting, locations, and relevant dates, including periods of recruitment, exposure, follow-up, and data collection	Methods ('Data sources' & 'Study design') & Figure 2
Participants	6	(*a*) Give the eligibility criteria, and the sources and methods of selection of participants. Describe methods of follow-up<br>(*b*) For matched studies, give matching criteria and number of exposed and unexposed	Methods ('Data sources' & 'Study design') & Figure 2
Variables	7	Clearly define all outcomes, exposures, predictors, potential confounders, and effect modifiers. Give diagnostic criteria, if applicable	Methods ('Study design', 'COVID-19 severity, criticality, and fatality classification', & 'Laboratory methods'), & Table 1
Data sources/ measurement	8*	For each variable of interest, give sources of data and details of methods of assessment (measurement). Describe comparability of assessment methods if there is more than one group	Methods ('Data sources', 'Study design', 'COVID-19 severity, criticality, and fatality classification', & 'Laboratory methods'), & Table 1
Bias	9	Describe any efforts to address potential sources of bias	Methods ('Study design', paragraph 5 & 'Statistical analysis')
Study size	10	Explain how the study size was arrived at	Figure 2
Quantitative variables	11	Explain how quantitative variables were handled in the analyses. If applicable, describe which groupings were chosen and why	Methods ('Study design', paragraphs 3 & 5, & 'Statistical analysis', paragraph 4) & Table 1
Statistical methods	12	(*a*) Describe all statistical methods, including those used to control for confounding	Methods ('Statistical analysis')
		(*b*) Describe any methods used to examine subgroups and interactions	NA
		(*c*) Explain how missing data were addressed	NA, see Methods ('Data sources')
		(*d*) If applicable, explain how loss to follow-up was addressed	NA, see Methods ('Data sources', & 'Study design', paragraph 1)
		(*e*) Describe any sensitivity analyses	Methods ('Statistical analysis', paragraph 6)
Results			
Participants	13*	(a) Report numbers of individuals at each stage of study—eg numbers potentially eligible, examined for eligibility, confirmed eligible, included in the study, completing follow-up, and analysed<br>(b) Give reasons for non-participation at each stage<br>(c) Consider use of a flow diagram	Results ('BA.1-against-BA.2 study', paragraphs 1-2 & 'BA.2-against-BA.1 study', paragraphs 1-2), Figure 2, & Table 1
Descriptive data	14	(a) Give characteristics of study participants (eg demographic, clinical, social) and information on exposures and potential confounders	Results ('BA.1-against-BA.2 study', paragraphs 1-2 & 'BA.2-against-BA.1 study', paragraphs 1-2), & Table 1
		(b) Indicate number of participants with missing data for each variable of interest	NA, see Methods ('Data sources', & 'Study design', paragraph 1)
		(c) Summarise follow-up time (eg, average and total amount)	Results ('BA.1-against-BA.2 study', paragraph 2 & 'BA.2-against-BA.1 study', paragraph 2), Figures 2 & 3, & Table 2
Outcome data	15	Report numbers of outcome events or summary measures over time	Results ('BA.1-against-BA.2 study', paragraphs 3-4 & 'BA.2-against-BA.1 study', paragraphs 3-4), Figures 2 & 3, & Table 2

Main results	16	(a) Give unadjusted estimates and, if applicable, confounder-adjusted estimates and their precision (eg, 95% confidence interval). Make clear which confounders were adjusted for and why they were included	Results ('BA.1-against-BA.2 study', paragraphs 3-4 & 'BA.2-against-BA.1 study', paragraphs 3-4), Figure 3, & Table 2
		(b) Report category boundaries when continuous variables were categorized	Table 1
		(c) If relevant, consider translating estimates of relative risk into absolute risk for a meaningful time period	NA
Other analyses	17	Report other analyses done—eg analyses of subgroups and interactions, and sensitivity analyses	Results ('BA.1-against-BA.2 study', paragraphs 5-6 & 'BA.2-against-BA.1 study', paragraphs 5-6)
Discussion			
Key results	18	Summarise key results with reference to study objectives	Discussion, paragraphs 1-2
Limitations	19	Discuss limitations of the study, taking into account sources of potential bias or imprecision. Discuss both direction and magnitude of any potential bias	Discussion, paragraphs 3-14
Interpretation	20	Give a cautious overall interpretation of results considering objectives, limitations, multiplicity of analyses, results from similar studies, and other relevant evidence	Discussion, paragraph 15
Generalisability	21	Discuss the generalisability (external validity) of the study results	Discussion, paragraph 14
Other information			
Funding	22	Give the source of funding and the role of the funders for the present study and, if applicable, for the original study on which the present article is based	Acknowledgements

Abbreviations: NA, not applicable.
