# Supplementary material for: Protection of Omicron sub-lineage infection against reinfection with another Omicron sub-lineage
Source: Nat Commun. 2022 Aug 9;13:4675. doi: 10.1038/s41467-022-32363-4 (PMC9362989; doi:10.1038/s41467-022-32363-4)
Supplement: Supplementary file 2 — Reporting Summary [file 41467_2022_32363_MOESM2_ESM.pdf]

nature research

Corresponding author(s): Laith J Abu-Raddad

Last updated by author(s): Jul 12, 2022

Reporting Summary

Nature Research wishes to improve the reproducibility of the work that we publish. This form provides structure for consistency and transparency in reporting. For further information on Nature Research policies, see our Editorial Policies and the Editorial Policy Checklist.

Statistics

For all statistical analyses, confirm that the following items are present in the figure legend, table legend, main text, or Methods section.

n/a	Confirmed	
☐	☒	The exact sample size (*n*) for each experimental group/condition, given as a discrete number and unit of measurement
☐	☒	A statement on whether measurements were taken from distinct samples or whether the same sample was measured repeatedly
☐	☒	The statistical test(s) used AND whether they are one- or two-sided<br>*Only common tests should be described solely by name; describe more complex techniques in the Methods section.*
☐	☒	A description of all covariates tested
☐	☒	A description of any assumptions or corrections, such as tests of normality and adjustment for multiple comparisons
☐	☒	A full description of the statistical parameters including central tendency (e.g. means) or other basic estimates (e.g. regression coefficient) AND variation (e.g. standard deviation) or associated estimates of uncertainty (e.g. confidence intervals)
☒	☐	For null hypothesis testing, the test statistic (e.g. *F*, *t*, *r*) with confidence intervals, effect sizes, degrees of freedom and *P* value noted<br>*Give P values as exact values whenever suitable.*
☒	☐	For Bayesian analysis, information on the choice of priors and Markov chain Monte Carlo settings
☒	☐	For hierarchical and complex designs, identification of the appropriate level for tests and full reporting of outcomes
☐	☒	Estimates of effect sizes (e.g. Cohen's *d*, Pearson's *r*), indicating how they were calculated

Our web collection on statistics for biologists contains articles on many of the points above.

Software and code

Policy information about availability of computer code

Data collection	No software was used for data collection
Data analysis	Analyses were conducted in STATA/SE 17.0. The code for pooling multiple imputed datasets can be found at: https://www.pharmasug.org/proceedings/2017/SP/PharmaSUG-2017-SP05.pdf

For manuscripts utilizing custom algorithms or software that are central to the research but not yet described in published literature, software must be made available to editors and reviewers. We strongly encourage code deposition in a community repository (e.g. GitHub). See the Nature Research guidelines for submitting code & software for further information.

Data

Policy information about availability of data

All manuscripts must include a data availability statement. This statement should provide the following information, where applicable:

- Accession codes, unique identifiers, or web links for publicly available datasets
- A list of figures that have associated raw data
- A description of any restrictions on data availability

The dataset of this study is a property of the Qatar Ministry of Public Health that was provided to the researchers through a restricted-access agreement that prevents sharing the dataset with a third party or publicly. The data are available under restricted access for preservation of confidentiality of patient data. Access can be obtained through a direct application for data access to Her Excellency the Minister of Public Health (https://www.moph.gov.qa/english/OurServices/eservices/Pages/Governmental-Health-Communication-Center.aspx). The raw data are protected and are not available due to data privacy laws. Data were available to authors through .csv files where information has been downloaded from the CERNER database system (no links/accession codes were available to authors). Aggregate data are available within the manuscript and its Supplementary information.

Field-specific reporting

Please select the one below that is the best fit for your research. If you are not sure, read the appropriate sections before making your selection.

☒ Life sciences ☐ Behavioural & social sciences ☐ Ecological, evolutionary & environmental sciences

For a reference copy of the document with all sections, see nature.com/documents/nr-reporting-summary-flat.pdf

Life sciences study design

All studies must disclose on these points even when the disclosure is negative.

Sample size	COVID-19 laboratory testing, vaccination, clinical infection data, and related demographic details were extracted from the integrated nationwide digital-health information platform that hosts the national, federated SARS-CoV-2 databases. These databases are complete and have captured all SARS-CoV-2-related data since epidemic onset. Cohorts were defined using national data that include every single individual tested using PCR in Qatar. To optimize specificity, the BA.1-infected and BA.2-infected cohorts were defined based on existence of an infection documented only using PCR and with a PCR cycle threshold value <30, between December 19, 2021 and March 21, 2022. Individuals previously infected, or who did not complete 35 days after Omicron BA.1/BA.2 infections or who died before the start of follow-up were excluded. The control cohorts were defined on the basis of PCR-negative tests between November 1, 2021 and December 18, 2021, to ensure that all persons in these cohorts have a record of a recent active residence in Qatar. Individuals previously infected or who died before the start of follow-up were excluded. Cohorts were exact-matched in a 1:1 ratio by sex, 10-year age group, nationality, and comorbidity count. Only matched cohorts were analyzed. Given that the sample sizes were based on national cohorts with only individuals that do not fit the eligibility criteria excluded, the sample size for each of the BA.1-against-BA.2 and BA.2-against-BA.1 studies can be considered sufficient. Detailed sample sizes can be found in Figure 2 and Table 1.
Data exclusions	Exclusion criteria for cohorts in each study were specified a priori. The BA.1-infected and BA.2-infected cohorts were defined based on existence of an infection documented only using PCR and with a PCR cycle threshold value <30, between December 19, 2021 and March 21, 2022. Individuals previously infected, or who did not complete 35 days after Omicron BA.1/BA.2 infections or who died before the start of follow-up were excluded. The control cohorts were defined on the basis of PCR-negative tests between November 1, 2021 and December 18, 2021, to ensure that all persons in these cohorts have a record of a recent active residence in Qatar. Individuals previously infected or who died before the start of follow-up were excluded.
Replication	For replication, multiple imputation analyses were conducted to estimate effectiveness of BA.1 infection against reinfection with BA.2 and effectiveness of BA.2 infection against reinfection with BA.1. There were minimal differences between the results of all 100 simulations and the pooled estimate generated following Rubin's rules. Two additional independent analyses were performed to estimate effectiveness 1) by adjusting the cox regression for time since vaccination at the start of the follow-up in addition to vaccination status and the matching factors and 2) by adjusting the estimates for differences in testing frequency between the cohorts. All analyses confirmed/reproduced estimates of effectiveness obtained in the main analysis.
Randomization	Not applicable as this is an observational cohort study where individuals are aware of both their infection status and their reinfection status. However, to ensure control of confounding, cohorts were exact-matched in a 1:1 ratio by sex, 10-year age group, nationality, and comorbidity count (Figure 2). To ensure that estimates of effectiveness of BA.1 infection against reinfection with BA.2 and effectiveness of BA.2 infection against reinfection with BA.1 were not biased, estimates were derived using a pooled measure for the hazard ratio obtained by pooling estimates across 100 datasets following Rubin's rules.
Blinding	Not applicable as this is an observational study cohort study where individuals are aware of both their infection and reinfection statuses.

Reporting for specific materials, systems and methods

We require information from authors about some types of materials, experimental systems and methods used in many studies. Here, indicate whether each material, system or method listed is relevant to your study. If you are not sure if a list item applies to your research, read the appropriate section before selecting a response.

Materials & experimental systems

n/a	Involved in the study	
☒	☐	Antibodies
☒	☐	Eukaryotic cell lines
☒	☐	Palaeontology and archaeology
☒	☐	Animals and other organisms
☐	☒	Human research participants
☒	☐	Clinical data
☒	☐	Dual use research of concern

Methods

n/a	Involved in the study	
☒	☐	ChIP-seq
☒	☐	Flow cytometry
☒	☐	MRI-based neuroimaging

Human research participants

Policy information about studies involving human research participants

Population characteristics	The demographic characteristics of the different study populations can be found in Table 1.
Recruitment	This is a retrospective study where COVID-19 laboratory testing, vaccination, clinical infection data, and related demographic details were extracted from the integrated nationwide digital-health information platform that hosts the national, federated SARS-CoV-2 databases. These databases are complete with no missing information for PCR testing, COVID-19 vaccinations, COVID-19 hospitalizations, comorbidity information, and basic demographic details, and have captured all SARS-CoV-2-related data since epidemic onset. The BA.1-infected and BA.2-infected cohorts were defined based on existence of an infection documented only using PCR and with a PCR cycle threshold value <30, between December 19, 2021 and March 21, 2022. Individuals previously infected, or who did not complete 35 days after Omicron BA.1/BA.2 infections or who died before the start of follow-up were excluded. The control cohorts were defined on the basis of PCR-negative tests between November 1, 2021 and December 18, 2021, to ensure that all persons in these cohorts have a record of a recent active residence in Qatar. Individuals previously infected or who died before the start of follow-up were excluded. All records of testing during the study duration were examined to identify eligible case and control cohorts as well as reinfections or infections with SARS-CoV-2.
Ethics oversight	The study was approved by the Hamad Medical Corporation and Weill Cornell Medicine-Qatar Institutional Review Boards with waiver of informed consent.

Note that full information on the approval of the study protocol must also be provided in the manuscript.
